# Real-world effectiveness of nirmatrelvir-ritonavir versus azvudine in hospitalized patients with COVID-19 during the omicron wave in Beijing: a multicenter retrospective cohort study

**DOI:** 10.1186/s12879-023-08965-8

**Published:** 2024-01-08

**Authors:** Xiaobo Han, Darui Gao, Chenglong Li, Xin Yuan, Junchang Cui, Weiguo Zhao, Fei Xie, Kaifei Wang, Yuhong Liu, Guoxin Muo, Na Xi, Mengli Zheng, Rentao Wang, Kun Xiao, Dahui Zhao, Xinxin Zhang, Xinjie Han, Bo Wang, Tiantian Zhang, Wuxiang Xie, Lixin Xie

**Affiliations:** 1 College of Pulmonary and Critical Care Medicine, The Eighth Medical Center, Chinese PLA General Hospital, No. 17 Heishanhu Road, Haidian District, Beijing, 100091 China; 2 Chinese PLA Medical School, Beijing, China; 3 Peking University Clinical Research Institute, Peking University First Hospital, No. 8 Xishiku Street, Xicheng District, Beijing, 100034 China; 4 Key Laboratory of Epidemiology of Major Diseases (Peking University), Ministry of Education, Beijing, China; 5 Pulmonary and Critical Care Medicine Department, The Fifth Medical Center, Chinese PLA General Hospital, Beijing, China; 6 Pulmonary and Critical Care Medicine Department, The First Medical Center, Chinese PLA General Hospital, Beijing, China; 7 Pharmacy Department, The Eighth Medical Center, Chinese PLA General Hospital, Beijing, China; 8 Shandong Future Network Research Institute, Jiangsu Future Network Group Co., Ltd., Nanjing, Jiangsu China

**Keywords:** Nirmatrelvir- ritonavir, Azvudine, COVID-19, SARS-CoV-2, Real-world effectiveness

## Abstract

**Background and aim:**

Two oral antivirals (Nirmatrelvir- ritonavir and Azvudine) are widely used in China practice during the Omicron wave of the pandemic. However, little evidence regarding the real-world effectiveness of these two oral antivirals in in-hospital patients. We aimed to evaluate the clinical effectiveness of nirmatrelvir-ritonavir versus azvudine among adult hospitalized patients with COVID-19.

**Methods:**

This retrospective cohort study used data from three Chinese PLA General Hospital medical centres. Hospitalized patients with COVID-19 treated with azvudine or nirmatrelvir-ritonavir from Dec 10, 2022, to February 20, 2023, and did not require invasive ventilation support on admission were eligible for inclusion.

**Results:**

After exclusions and propensity-score matching, the final analysis included 486 azvudine recipients and 486 nirmatrelvir-ritonavir recipients. By 28 days of initiation of the antivirus treatment, the crude incidence rate of all-cause death was similar in both types of antivirus treatment (nirmatrelvir-ritonavir group 2.8 events 1000 person-days [95% CI, 2.1–3.6] vs azvudine group 3.4 events/1000 person-days [95% CI, 2.6–4.3], *P* = 0.38). Landmark analysis showed that all-cause death was lower in the nirmatrelvir-ritonavir (3.5%) group than the azvudine (6.8%, *P* = 0.029) within the initial 10-day admission period, while no significant difference was observed for results between 10 and 28 days follow-up. There was no significant difference between the nirmatrelvir-ritonavir group and the azvudine group in cumulative incidence of the composite disease progression event (8.6% with nirmatrelvir-ritonavir vs. 10.1% with azvudine, HR, 1.22; 95% CI 0.80–1.86, *P* = 0.43).

**Conclusion:**

Among patients hospitalized with COVID-19 during the omicron wave in Beijing, similar in-hospital clinical outcomes on 28 days were observed between patients receiving nirmatrelvir-ritonavir and azvudine. However, it is worth noticing that nirmatrelvir-ritonavir appears to hold an advantage over azvudine in reducing early mortality. Further randomized controlled trials are needed to verify the efficacy of those two antivirus medications especially in early treatment.

**Supplementary Information:**

The online version contains supplementary material available at 10.1186/s12879-023-08965-8.

## Introduction

In the context of the deadly COVID-19 pandemic, various developed or repurposed oral antiviral medications have been authorized for emergency use to treat SARS-CoV-2 infection [1, 2]. Nirmatrelvir-ritonavir and azvudine are the main antivirus medications granted emergency approval by the Chinese National Drug Administration. During the Omicron wave in China that began at the end of 2022, those two antivirus medications are both recommended by the National Health Commission guidelines for treating adult patients with mild to moderate COVID-19 to reduce viral load and thereby prevent disease progression and death [3]. However, whether there is a difference in the effectiveness of clinical outcomes between azvudine and nirmatrelvir-ritonavir among hospitalized patients with COVID-19 is still unclarified.

Accumulated evidence from a large randomized controlled trial and cohort studies showed consistent results that nirmatrelvir-ritonavir for nonhospitalized adults at high risk of disease progression within 5 days of symptom onset could effectively reduce COVID-19-related hospitalization or death [4–7]. Nirmatrelvir-ritonavir is believed to be a better choice for antivirus treatment because more evidence supports the efficiency of clinical benefits and reduction of viral load [8, 9]. However, there remain controversial clinical outcomes among hospitalized patients with different respiratory support [8, 10].

Azvudine is a broad-spectrum small nucleoside analog inhibitor developed initially for treating HIV infection in China and is currently used to reduce the replication of SARS-CoV-2 [11, 12]. Two phase III randomized controlled trials in Brazil found that azvudine may shorten the time to negative nucleic acid conversion and reduce treatment time among mild and moderate COVID-19 patients compared to those who received regular care [13, 14]. Due to the few interactions with other medications and the lack of limitations on the time of symptom onset, azvudine was widely used during the Omicron wave in China. Regardless of the widespread usage in practice, few evidence supporting the clinical effectiveness of azvudine treatment is established in the real-world or possesses an advantage of the multicenter design [15, 16].

Given the ever-sustaining severe challenges posed by COVID-19 to the healthcare system, the comprehensive comparison of the potential clinical effectiveness between available treatment options would certainly be relevant for the current practice. Therefore, we conducted this retrospective cohort study to compare the effectiveness of oral azvudine and nirmatrelvir-ritonavir in hospitalized patients with COVID-19 during the Omicron wave in China.

## Methods

### Study design and participants

This retrospective cohort study was conducted at three medical centers of PLA General Hospital (the First, the Fifth, and the Eighth medical centers, Beijing) under the Chinese PLA General Hospital Group, which is located in different districts of Beijing, China. The Ethics Committee of PLA General Hospital approved all data analyses and exempted informed consent requirements on account of the minimal risk of this retrospective cohort study (number 309202302230712). This study was conducted following the Strengthening the Reporting of Observational Studies in Epidemiology (STROBE) reporting guidelines [17]. No compensation was offered for participating in this study.

Patients were eligible for our study if they were admitted with confirmed COVID-19 for the first time between December 10, 2022, and February 20, 2023, and were treated with azvudine or nirmatrelvir-ritonavir alone. Patients were excluded from the analysis if they were less than 18 years old, discharged or died within 24 hours after being prescribed antiviral treatment, or required invasive mechanical ventilation at baseline.

### Data collection

The data were derived from the three medical centers of the Chinese PLA General Hospital with inpatient and outpatient electronic medical records. Clinical researchers and engineers collaborated to build a database of COVID-19 inpatients of medical centers of PLA during the study periods. Data included demographics, vital signs, laboratory tests, previous medical history, nursing records, diagnoses, and inpatient orders. The pharmacy’s electronic records were used to verify the actual dosage of the prescribed order. Data on survival after discharge were obtained by telephone interviews. The final follow-up date was March 22, 2023. Two clinical doctors verified the availability of the database. Data were accessible through a secure platform hosted by the hospital server within a private virtual network.

### Baseline covariates

We extracted variables, including age, sex, time from symptom onset to initiation of antivirus, laboratory test results, clinical severity, corticosteroid therapy, nosocomial infection, and comorbidities. Comorbidities were categorized according to the Charlson comorbidity index (19 items of different medical comorbid conditions with different clinical weights to predict long-term mortality) as 0, 1–3, and above 3 [18]. Nosocomial infection was defined as the original inpatients that hospitalization before COVID-19 diagnosis. The baseline was the day that the patient started receiving nirmatrelvir-ritonavir or azvudine for the first time during hospitalization. The baseline laboratory test results included white blood cell count (WBC), hemoglobin, lymphocyte count, platelet count, albumin, alanine aminotransferase, estimated glomerular filtration rate (eGFR), C-reactive protein (CRP), and D-dimer on the day of baseline or within 48 hours before and after baseline (if the baseline-day values were missing). The World Health Organization (WHO) 8-point ordinal scale was assessed daily until 28 days of hospitalization or discharge to evaluate the severity or clinical severity [19]. The range of this scale was from 3 (no oxygen required) to 8 (death).

### Treatment exposure

All three medical centers had equal access to nirmatrelvir-ritonavir or azvudine during the study periods. The therapeutic strategies are based on the expert consensus from the Respiratory Society of the Chinese Medical Association and Guidelines for the Diagnosis and Treatment of COVID-19 in China [3, 20]. The criteria for the use of nirmatrelvir-ritonavir and azvudine in the patients recommended by the guideline and consensus are essentially the same. Both two medications are recommended for prescribed in patients with mild to moderate COVID-19 who are at high risk of progressing to critical illness. In addition, for safety reasons, nirmatrelvir-ritonavir should not be used in certain patients whose concomitant medications are dependent on CYP3 for clearance, whereas azvudine does not have those restrictions. Doctors prescribed oral antivirals to patients with COVID-19 as clinically appropriate based on the recommended of criteria. Patients who were prescribed nirmatrelvir-ritonavir after admission and did not receive azvudine or any other antiviral medications throughout their hospitalization were considered nirmatrelvir-ritonavir therapeutic exposure. Similarly, patients who were prescribed azvudine admission and did not receive nirmatrelvir-ritonavir or any other antiviral medication during their hospital stay were considered exposed to azvudine treatment. The recommended dose for nirmatrelvir-ritonavir was 300 mg nirmatrelvir and 100 mg ritonavir twice per day for 5 days or less and azvudine was 5 mg once a day for < 14 days.

### Outcomes

The primary outcome was all-cause death recorded within 28 days. The secondary outcome was a composite outcome of disease progression (new need for invasive mechanical ventilation or death during 28 days). The other assessment included the rate of clinical improvement and the median time of clinical improvement. Clinical improvement was defined as a decrease of 2 points on the WHO 8-point ordinal scale within 28 days of hospitalization or at hospital discharge with no increase in score from the baseline score (whichever occurred first). Adverse events included severe liver and kidney function impairment (defined as the new onset of an eGFR < 30 ml/min/1.73 m^2^) and alanine aminotransferase concentrations > 200 U/L during hospitalization) [21].

### Statistical analysis

The patients’ characteristics are shown as the mean ± standard deviation or the median with interquartile range for continuous variables and as number (%) for categorical variables. Differences in baseline characteristics were tested using Student’s t-test, the Wilcoxon rank test, or the chi-square test.

We performed 1:1 propensity score matching to account for the observed imbalance in covariates between the nirmatrelvir-ritonavir and azvudine groups. Binary logistic regression was applied to estimate the conditional probability of receiving nirmatrelvir-ritonavir after admission. The covariables used for matching included age, sex, time from symptom onset to admission, the Charlson comorbidity index, eGFR, CRP, WBC, WHO 8-point ordinal scale at baseline, corticosteroid therapy, complications (e.g., chronic liver disease, tumors, diabetes), and original inpatient, which was determined based on professional knowledge and previous reports [8, 21]. The caliper matching algorithm without replacement was used, and a caliper value of 0.2 was applied to conduct the matching procedure. The Love plot showing absolute standardized mean differences was used for evaluating the balance of covariates before and after propensity-score matching, with a threshold of 0.1 applied for determining imbalance [22]. All analyses were conducted based on matched samples.

The primary analysis aimed to evaluate the associations between treatment exposure and study during hospitalization. We recorded various outcomes, such as all-cause death, composite disease progression within 28 days, and clinical improvement. The duration between the initiation of antivirus medication after admission and the occurrence of events, discharge date, or date of death (whichever occurred first) was considered the time to study outcome. We used the Kaplan–Meier estimator and applied the log-rank test to detect potential differences. Additionally, we calculated the cumulative incidence of study outcomes. We also calculated the crude incidence rate per 1000 person-days of outcomes. To provide insight into the difference in the early and late cumulative incidence rates of all-cause death and composite disease progression outcome in patients with different treatments, we performed a “landmark survival analysis” with a landmark timepoint specified at 10 days [23, 24]. With regard to study outcomes, Cox proportional hazard regression was used to estimate the hazard ratio (HR) and 95% confidence interval (CI) associated with the treatment exposure. We accounted for the heterogeneity between study hospitals by adding a frailty term to the Cox models [25].

Several sensitivity analyses were also conducted. First, we performed a stratified analysis according to the duration from symptom onset to treatment initiation (≤5 days and >5 days). Second, we performed subgroup analyses according to selected baseline characteristics and applied the z-test to detect potential modifying factors [26]. Third, we compared safety outcomes between the groups and used the Wald method to calculate the risk ratio.

All statistical analyses were conducted using SAS software, version 9.4 (SAS Institute Inc.) and R language 3.4.0 (R Foundation, Vienna, Austria). A two-tailed alpha of 0.05 indicates a statistically significant level.

### Role of the funding source

The funders were not involved in the study design, collection, analysis, or interpretation of data or report writing.

## Results

In the study, we identified 4201 consecutive hospitalized adults in the PLA healthcare system diagnosed with COVID-19. Among those who met the inclusion and exclusion criteria, 563 received azvudine, and 909 received nirmatrelvir-ritonavir. After propensity score matching, 486 patients in the azvudine group and 486 in the nirmatrelvir-ritonavir group were included in the analysis (Appendix 1, supplementary Fig. 1). Table 1 shows the baseline characteristics of patients in the azvudine and the nirmatrelvir-ritonavir groups before and after 1:1 propensity score matching. Before matching, patients in the azvudine group were younger, had a higher proportion of chronic lung disease, and had a lower proportion of tumors than those in the nirmatrelvir-ritonavir group. Baseline covariates were balanced after matching, with a corresponding absolute standardized mean difference < 0.1 (Appendix 1, supplementary Fig. 2). The median time from symptom onset to initiating antiviral therapy was 8.0 days (95% CI: 4.0–13.0), with 64.3% of patients initiating antiviral treatment 5 days after the onset of symptoms. A total of 81.9% of patients had at least one comorbidity in this study.

**Table 1:** Baseline characteristics of participants before and after propensity-score matching

Characteristics ^a^	Before matching			After 1:1 propensity-score matching		
	nirmatrelvir-ritonavir (*n* = 909)	azvudine (*n* = 563)	*P* for difference ^b^	nirmatrelvir-ritonavir (*n* = 486)	azvudine (*n* = 486)	*P* for difference ^b^
Age, mean years	72.4 (17.8)	64.4 (22.0)	< 0.001	67.9 (17.4)	68.0 (19.9)	0.947
Sex (male)	592 (65.1%)	352 (62.5%)	0.339	315 (64.8%)	307 (63.2%)	0.64
Symptoms onset to exposure	8.0 [4.0–13.0]	7.5 [3.0–12.0]	0.087	8.0 [4.0–13.0]	8.0 [4.0–13.0]	0.959
≤5 days	319 (35.1%)	225 (40.0%)		176 (36.2%)	177 (36.4%)	
>5 days	570 (62.7%)	329 (58.4%)	0.142	303 (62.3%)	301 (61.9%)	0.963
Unclear	20 (2.2%)	9 (1.6%)		7 (1.4%)	8 (1.6%)	
Respiratory support						
No oxygen therapy required	165 (34.0%)	164 (33.7%)		165 (34.0%)	164 (33.7%)	
Requiring oxygen therapy	292 (60.1%)	289 (59.5%)	0.871	292 (60.1%)	289 (59.5%)	0.871
Requiring HFNC or NPPV	29 (6.0%)	33 (6.8%)		29 (6.0%)	33 (6.8%)	
WHO 8-point ordinal scale	3.7 ± 0.5	3.7 ± 0.6	0.079	3.7 ± 0.6	3.7 ± 0.6	0.779
Charlson comorbidity index	1.0 [0.0–3.0]	1.0 [0.0–3.0]	0.118	2.0 [0.0–3.0]	1.0 [0.0–3.0]	0.354
Pre-existing comorbid conditions						
Malignant tumors	195 (21.5%)	88 (15.6%)	0.007	98 (20.2%)	85 (17.5%)	0.325
kidney transplantation	50 (5.5%)	23 (4.1%)	0.275	39 (8.0%)	21 (4.3%)	0.023
chronic liver disease	125 (13.8%)	98 (17.4%)	0.068	86 (17.7%)	83 (17.1%)	0.866
diabetes	267 (29.4%)	147 (26.1%)	0.196	149 (30.7%)	139 (28.6%)	0.527
chronic kidney disease	117 (12.9%)	111 (19.7%)	0.001	69 (14.2%)	96 (19.8%)	0.026
hypertension	445 (49.0%)	249 (44.2%)	0.087	233 (47.9%)	231 (47.5%)	0.949
cardiovascular disease	258 (28.4%)	143 (25.4%)	0.234	121 (24.9%)	133 (27.4%)	0.422
cerebrovascular diseases	153 (16.8%)	74 (13.1%)	0.067	79 (16.3%)	69 (14.2%)	0.422
dementia	40 (4.4%)	18 (3.2%)	0.31	20 (4.1%)	17 (3.5%)	0.737
connective tissue disease	29 (3.2%)	12 (2.1%)	0.3	16 (3.3%)	11 (2.3%)	0.435
Nosocomial infection	114 (12.5%)	81 (14.4%)	0.349	66 (13.6%)	70 (14.4%)	0.781
Laboratory results, mean (SD)						
White blood cell count, 10^9^ cells/ L	5.8 [4.1–8.1]	5.8 [4.3–8.1]	0.745	5.7 [4.1–8.5]	5.8 [4.3–8.2]	0.686
Lymphocyte count, 10^9^ cells/ L	0.9 [0.6–1.2]	0.8 [0.5–1.3]	0.515	0.9 [0.5–1.3]	0.8 [0.5–1.2]	0.663
Hemoglobin, g/L	119.8 ± 27.5	122.1 ± 25.5	0.099	121.1 ± 27.9	120.8 ± 24.6	0.819
Albumin, g/L	34.5 ± 5.8	35.5 ± 6.8	0.004	35.1 ± 6.0	35.0 ± 6.4	0.686
Alanine aminotransferase, U/L	22.1 [14.1–36.0]	20.0 [13.6–31.8]	0.003	23.0 [14.4–36.0]	20.0 [14.0–31.0]	0.021
CRP, mg/L	22.9 [7.0–59.4]	22.3 [6.0–60.1]	0.934	23.7 [7.1–63.3]	23.1 [6.0–59.8]	0.481
eGFR	88.5 [67.0–109.5]	95.0 [64.6–119.7]	0.034	91.5 [67.2–115.1]	93.9 [63.4–119.6]	0.721
Concomitant medications, No. (%)						
Corticosteroids use	390 (42.9%)	259 (46.0%)	0.267	228 (46.9%)	237 (48.8%)	0.607
Tocilizumab use	44 (4.8%)	23 (4.1%)	0.584	28 (5.8%)	23 (4.7%)	0.565

*eGFR* estimated Glomerular Filtration Rate, *CRP* C-reactive protein, *CCI* Charlson comorbidity index, *HFNC* High Flow Nasal Cannula therapy, *NPPV* Non-invasive Positive Pressure Ventilation, *WHO* World Health Organization

^a^Data are presented as mean ± SD, n (%), or median (quartile 1-quartile 3)

^b^*P* value reported for differences between two cohorts using t-test, chi-square test, or Wilcoxon rank test

**Fig. 1:**
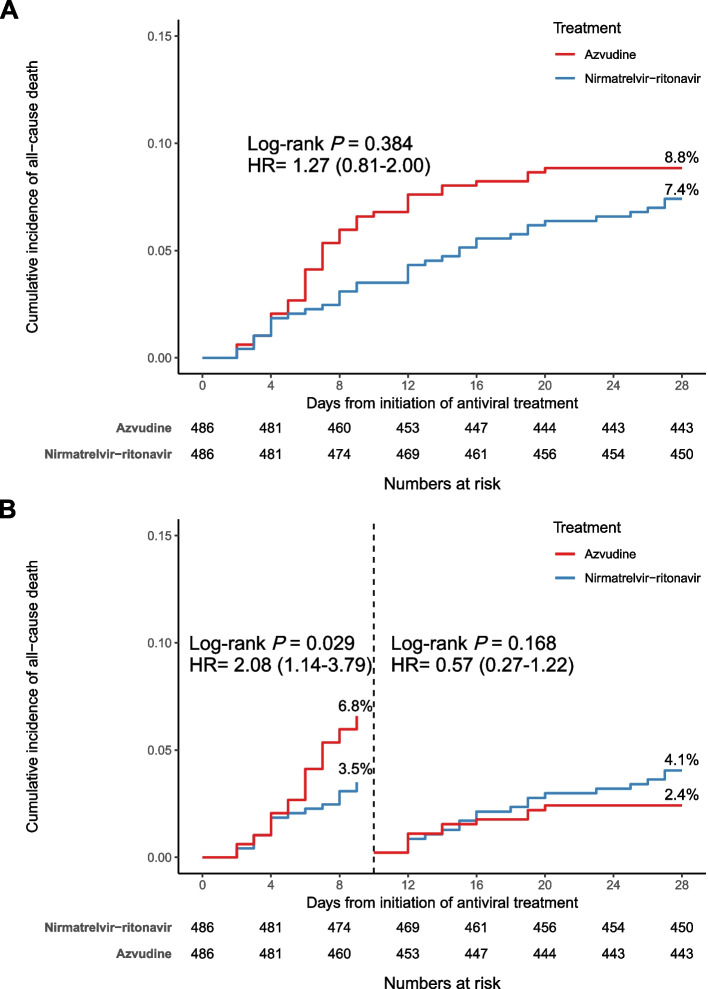
Cumulative incidence of all-cause death for nirmatrelvir-ritonavir group vs azvudine group. **A** Cumulative incidence of all-cause death in the nirmatrelvir-ritonavir group and azvudine group. **B** Landmark analysis discriminating between all-cause death events occurring before and after 10 days of follow-up. Day 0 (baseline) represents the first day of initiating antivirus treatment. The Kaplan–Meier estimator was used to estimate cumulative incidence, with the log-rank test applied to assess differences between groups

By 28 days of initiation of antivirus treatment, the crude rate of all-cause death in the azvudine group was 3.4 (95% CI, 2.6–4.3) events per 1000 person-days and 2.8 (95% CI: 2.1–3.6) events per 1000 person-days in the nirmatrelvir-ritonavir group (HR for death, 1.27; 95% CI, 0.81 to 2.00, *P* = 0.384; Table 2, Fig. 1). Landmark analysis discriminating between events occurring before and after 10 days of initiation of the antivirus treatment showed that the risk of all-cause death was higher in the azvudine group than in the nirmatrelvir-ritonavir group (rate of 6.8% vs 3.5%, HR, 2.08 (1.14–3.79), *P* = 0.029) by 10 days. There was no difference in this result after 10 days of follow-up (nirmatrelvir-ritonavir 4.1% vs azvudine 2.4%, *p* = 0.168, Fig. 1).

**Table 2:** Study outcomes in nirmatrelvir-ritonavir group vs. azvudine

Outcomes	Crude incidence (%)	Person-days	Crude incidence rate per 1000 person-days (95% CI)	HR (95% CI) ^a^
**All-cause death** ^**b**^				
Nirmatrelvir-ritonavir	36 (7.4%)	13,030	2.8 (2.1–3.6)	Reference
Azvudine	43 (8.8%)	12,746	3.4 (2.6–4.3)	1.27 (0.81–2.00)
**Composite disease progression** ^**c**^				
Nirmatrelvir-ritonavir	42 (8.6%)	12,804	3.3 (2.5–4.2)	Reference
Azvudine	49 (10.1%)	12,530	3.9 (3.1–4.9)	1.22 (0.80–1.86)
**Clinical improvement** ^d^				
Nirmatrelvir-ritonavir	376 (77.4%)	6818	55.1 (50.8–59.9)	Reference
Azvudine	389 (80.0%)	6366	61.1 (56.4–66.2)	1.10 (0.95–1.27)

*HR* hazard ratio, *CI* confidence interval

^a^HR was estimated using Cox proportional hazard regression

^b^Defined as all-cause mortality recorded within 28 days after exposure

^c^Defined as new incidents of invasive mechanical ventilation or death within 28 days after exposure

^d^Defined as 2 points or higher improvement in clinical symptoms recorded within 28 days after exposure

**Fig. 2:**
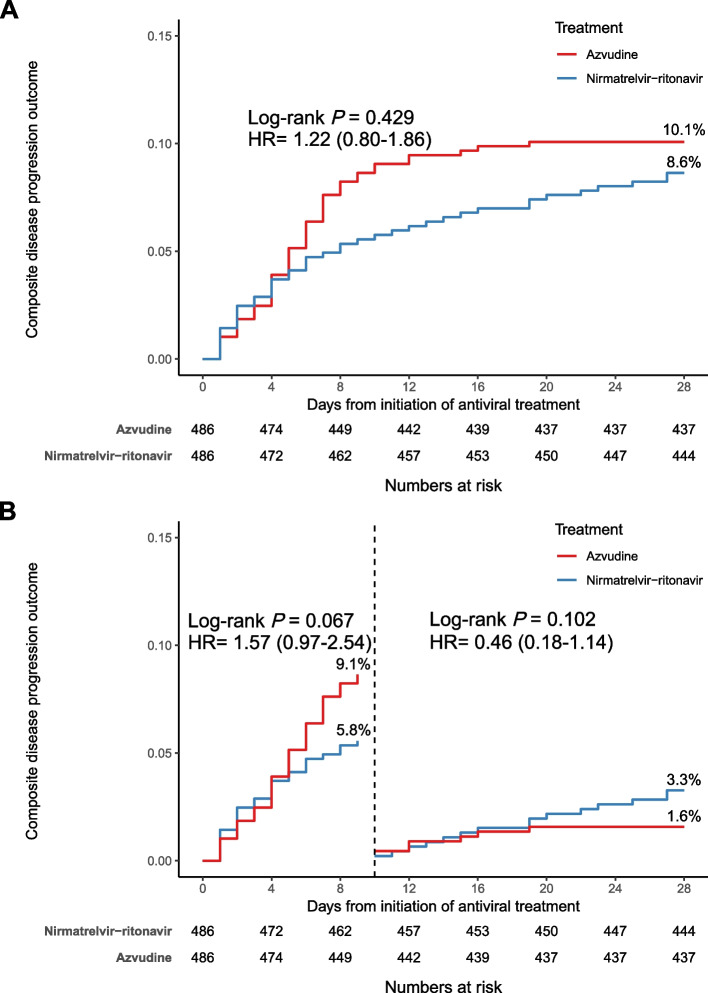
Cumulative incidence of composite disease progression events for nirmatrelvir-ritonavir group vs azvudine group. **A** Cumulative incidence of composite disease progression (new incidents of invasive mechanical ventilation or death during 28 days of follow-up) in the nirmatrelvir-ritonavir and azvudine groups. **B** Landmark analysis discriminating between composite disease progression events occurring before and after 10 days of follow-up

The rate for composite disease progression by 28 days in the azvudine group was 10.1% compared with the nirmatrelvir-ritonavir group at 8.6% (HR, 1.22; 95% CI, 0.80 to 1.86, *P* = 0.429; Table 2, Fig. 2). There was no difference in Landmark analysis discriminating between events occurring before and after 10 days in the two groups (Fig. 2).

The cumulative clinical improvement rate in the azvudine group on the 28th hospital day was 80.0%, and the median time to improvement was 11.0 days (95% CI, 11.0–12.0). The cumulative clinical improvement rate on the 28th hospital day in the nirmatrelvir-ritonavir group was 77.4%, and the median time to clinical improvement was 12.0 (11.0–14.0) days. There was no difference in clinical improvement between the two groups (Table 2, Appendix 1, supplemental Fig. 3).

The severe adverse events including severe injury to the kidney or liver were similar as shown in supplemental Table 1, supplemental Table 1. Specifically, the incidence of newly onset severe kidney injury was 3.3% in the nirmatrelvir-ritonavir group and 2.7% in the azvudine group (RR, 0.89; 95% CI,0.59–1.34, *P* = 0.579). The incidence of newly onset severe liver injury was 3.5% in the nirmatrelvir-ritonavir group and1.9% in the azvudine group (RR, 0.69; 95% CI, 0.40–1.17, *P* = 0.117).

### Sensitivity analyses

**Fig. 3:**
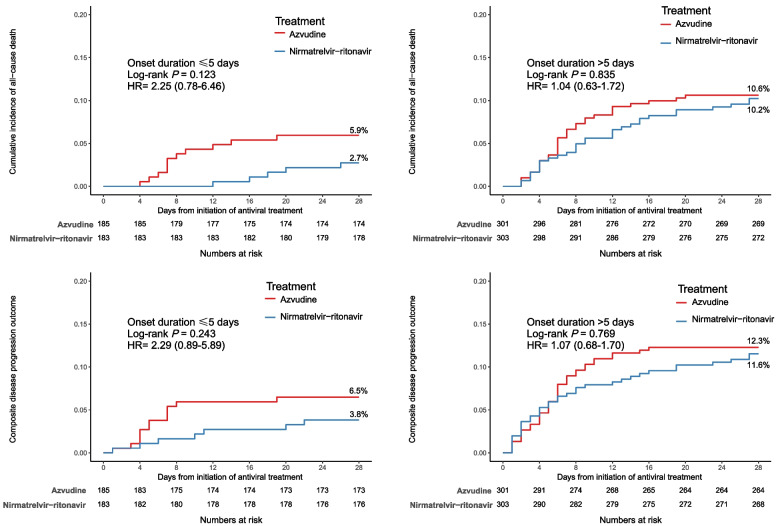
Cumulative incidence of study outcomes for nirmatrelvir-ritonavir recipients vs azvudine recipients, further stratified by duration from symptom onset to treatment initiation

The results of the subgroup analysis are consistent with those of the primary analysis. There were no significant differences in the risk of all-cause death, composite disease progression between the two groups stratified according to the duration from symptom onset to treatment initiation (≤5 days and >5 days) (Fig. 3). In addition, subgroup analysis of all-cause death events showed that recipients of azvudine had higher mortality within 28 after exposure in those with hypertension than those of nirmatrelvir-ritonavir (P for interaction = 0.032, Appendix 1, supplemental Fig. 4). Additionally, the subgroup analysis of composite disease progression showed that recipients of azvudine had higher events within 28 days after exposure in those with chronic liver disease than those of nirmatrelvir-ritonavir (P for interaction =0.043, Appendix 1, supplemental Fig. 5).

## Discussion

This is the first multicenter real-world study that compares the effectiveness of oral azvudine and nirmatrelvir-ritonavir in hospitalized patients with COVID-19 head-to-head during the Omicron wave in Beijing. We found no significant differences in clinical outcomes, including all-cause death, composite disease progression, and clinical improvement at day 28 between oral azvudine and nirmatrelvir-ritonavir.

Previous studies have identified consistent conclusions about the effectiveness of nirmatrelvir-ritonavir in mild patients with COVID-19, while a large proportion of patients in the real-world setting are exceeding the indications for nirmatrelvir-ritonavir treatment during the Omicron wave in Beijing, China. Azvudine is urgently used as a candidate antivirus medication in this context. Although in a small cohort study, nirmatrelvir-ritonavir was superior to azvudine in terms of more rapid virus suppression and an earlier RT–PCR negative conversion, evidence regarding the difference in effectiveness in clinical outcomes between nirmatrelvir-ritonavir and azvudine treatment in hospitalized patients with COVID-19 is insufficient and from a single center [10, 15, 16]. In this study, 60% of patients at baseline required oxygen, and 6.4% received high-flow cannula oxygen therapy or noninvasive mechanical ventilation. In addition, more than 60% of patients did not start antiviral treatment within 5 days of symptom onset. The population in this study is quite different from those in previous studies with mild to moderate COVID-19 patients, which also reflects the reality during this study period [7, 8]. Old patients with delayed antivirus treatment and multiple comorbidities may be one of the possible reasons for the high incidence of death and the delay in achieving improvement in this study.

Notably, more than half of the death events occurred within 10 days of initiating antivirus treatment in this study. Therefore, we performed a landmark analysis and found a significantly higher risk of death during the first 10 days in patients who received azvudine than in patients who received nirmatrelvir-ritonavir. After the first 10 days, the risk of all-cause death was similar in those two antivirus groups. Thus, the difference in mortality might occur mainly within the early stage after antivirus treatment as an acute infectious disease. The association between nirmatrelvir-ritonavir and death events might be attenuated when the clinical course is sustained. The clinical benefit of nirmatrelvir-ritonavir in reducing early death in hospitals might provide more opportunities for treatment.

Another feature of this study is that more than 60% of the patients had exceeded the recommended time for treatment within 5 days of symptom onset. As such, we proceeded with subgroup analyses based on whether antiviral therapy commenced within 5 days of symptom onset. Although the clinical outcomes regarding all causes of death, composite disease progression and clinical improvement were similar in this subgroup, we also observed that patients with nirmatrelvir-ritonavir had a trend of lower mortality compared to patients with azvudine when used within 5 days of symptoms onset. Considering the limited number of patients who initiate antiviral treatment within 5 days of onset in our study and the importance of early antiviral treatment in evaluating the effectiveness of antiviral medications, future studies need to focus on the early efficacy of the two antiviral medications.

Although this is the first study that includes severe patients requiring oxygen and intensive respiratory support in a multicenter, real-world setting to compare the effectiveness of azvudine and nirmatrelvir-ritonavir among COVID-19 inpatients, there are several limitations to this retrospective cohort study. First, although covariates at baseline were well balanced after matching, vaccination status was not adjusted owing to the large proportion of missing values. With a full vaccination rate of more than 90% in China, adjusting for vaccines may have had little impact on the results [27]. Second, the time of symptom onset might be inaccurate since some COVID-19 symptoms were not obvious. We tried to minimize this bias by matching the time of symptom onset as a categorical covariate. Third, given the small number of patients who initiated antiviral therapy within 5 days of symptom onset in the subgroup analysis, the results should be interpreted with caution.

In summary, this multicenter, retrospective cohort study demonstrated that clinical outcomes within 28 days were similar for azvudine and nirmatrelvir-ritonavir among hospitalized patients with COVID-19 during the Omicron wave in Beijing. While recipients of nirmatrelvir-ritonavir had lower mortality than the azvudine group at 10 days, the difference was of uncertain clinical explanation. Further randomized controlled trials are needed to verify these findings.

### Supplementary Information

**Additional file 1.**

## Data Availability

The data custodians (the Chinese PLA General Hospital) provided the underlying individual-patient data only for the study of this project. Data access was under the authorization of the confidentiality committees. The data and materials supporting the conclusions of the study are available from the corresponding author (E-mail: xielx301@126.com) upon reasonable request.

## Abbreviations

CRP: C-reactive protein
CI: Confidence interval
COPD: Chronic obstructive pulmonary disease
CRD: Chronic respiratory disease
eGFR: estimated Glomerular Filtration Rate
HR: Hazard ratios
HFNC: High Flow Nasal Cannula therapy
NPPV: Non-invasive Positive Pressure Ventilation
SD: Standard deviation
WHO: World Health Organization
WBC: White blood cell count
