# Supplementary material for: Real-world effectiveness of nirmatrelvir-ritonavir versus azvudine in hospitalized patients with COVID-19 during the omicron wave in Beijing: a multicenter retrospective cohort study
Source: BMC Infect Dis. 2024 Jan 8;24:57. doi: 10.1186/s12879-023-08965-8 (PMC10773102; doi:10.1186/s12879-023-08965-8)
Supplement: Supplementary file 1 — Additional file 1. [file 12879_2023_8965_MOESM1_ESM.docx]

**Supplemental Tables**

[**Supplemental Table 1.** Comparison of safety outcomes between nirmatrelvir–ritonavir group and azvudine group.](#_Toc135592074)

**Supplemental Figures**

**Supplemental Figure 1.** Participants selection diagram.

**Supplemental Figure 2.** Love plot assessing differences in baseline characteristics before and after 1:1 propensity-score matching.

Absolute standardized mean differences were applied to assess covariate balance, with a threshold of > 0.1 used to determine imbalance.

^a^CCI, Charlson Comorbidity Index.

**Supplemental Figure 3. Cumulative incidence of clinical improvement events for nirmatrelvir–ritonavir（Paxlovid） group vs azvudine group.**

Day 0 (baseline) represents the ﬁrst day of initiating antivirus treatment. The Kaplan‒Meier estimator was used to estimate cumulative incidence, with the log-rank test applied to assess differences between groups.

**Supplemental Figure 4.** Subgroup analysis comparing all-cause mortality risks between the nirmatrelvir–ritonavir (Paxlovid) group and the azvudine group, as defined according to baseline characteristics.

**Supplemental Figure 5.** Subgroup analysis comparing incidences of composite disease progression between the nirmatrelvir–ritonavir (Paxlovid) group and the azvudine group, as defined according to baseline characteristics.

**Supplemental Table 1. Comparison of safety outcomes between nirmatrelvir–ritonavir group and azvudine group.**

| **Safety outcomes** | **Cumulative incidence** | **RR (95% CI) ^a^** | ***P*** |
| --- | --- | --- | --- |
| **Severe liver impairment** |  |  |  |
| Nirmatrelvir–ritonavir | 16 (3.3%) | Reference | / |
| Azvudine | 13 (2.7%) | 0.89 (0.59-1.34) | 0.579 |
| **Severe renal impairment** |  |  |  |
| Nirmatrelvir–ritonavir | 17 (3.5%) | Reference | / |
| Azvudine | 9 (1.9%) | 0.69 (0.40 - 1.17) | 0.117 |

RR: risk ratio; CI: confidence interval.

^a^ RR was calculated using the Wald method.

**
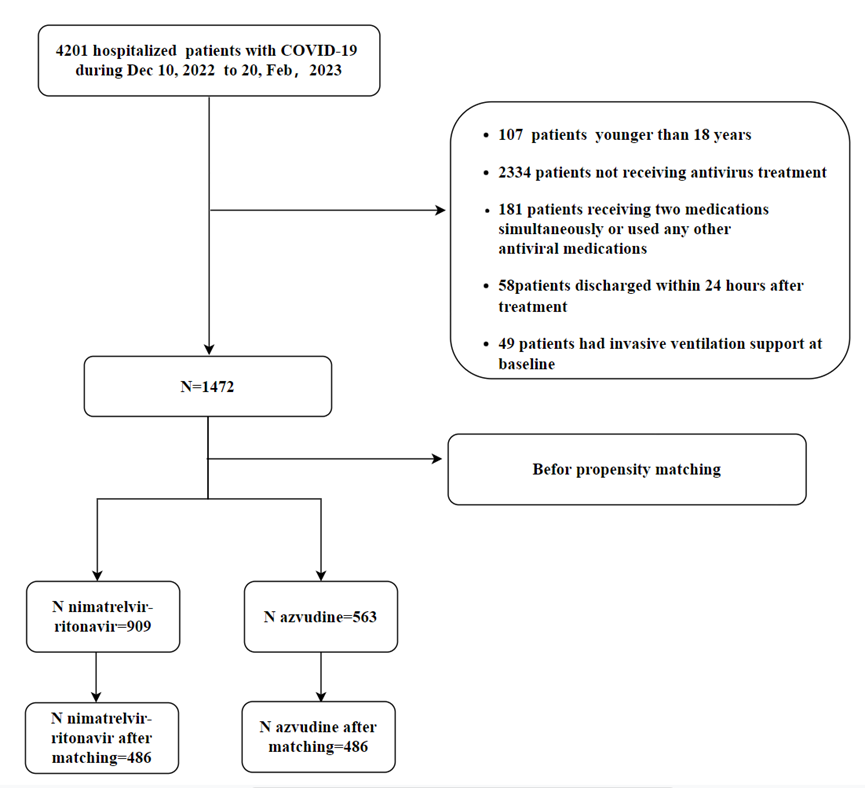
**

**Supplemental Figure 1. Participants selection diagram.**

**
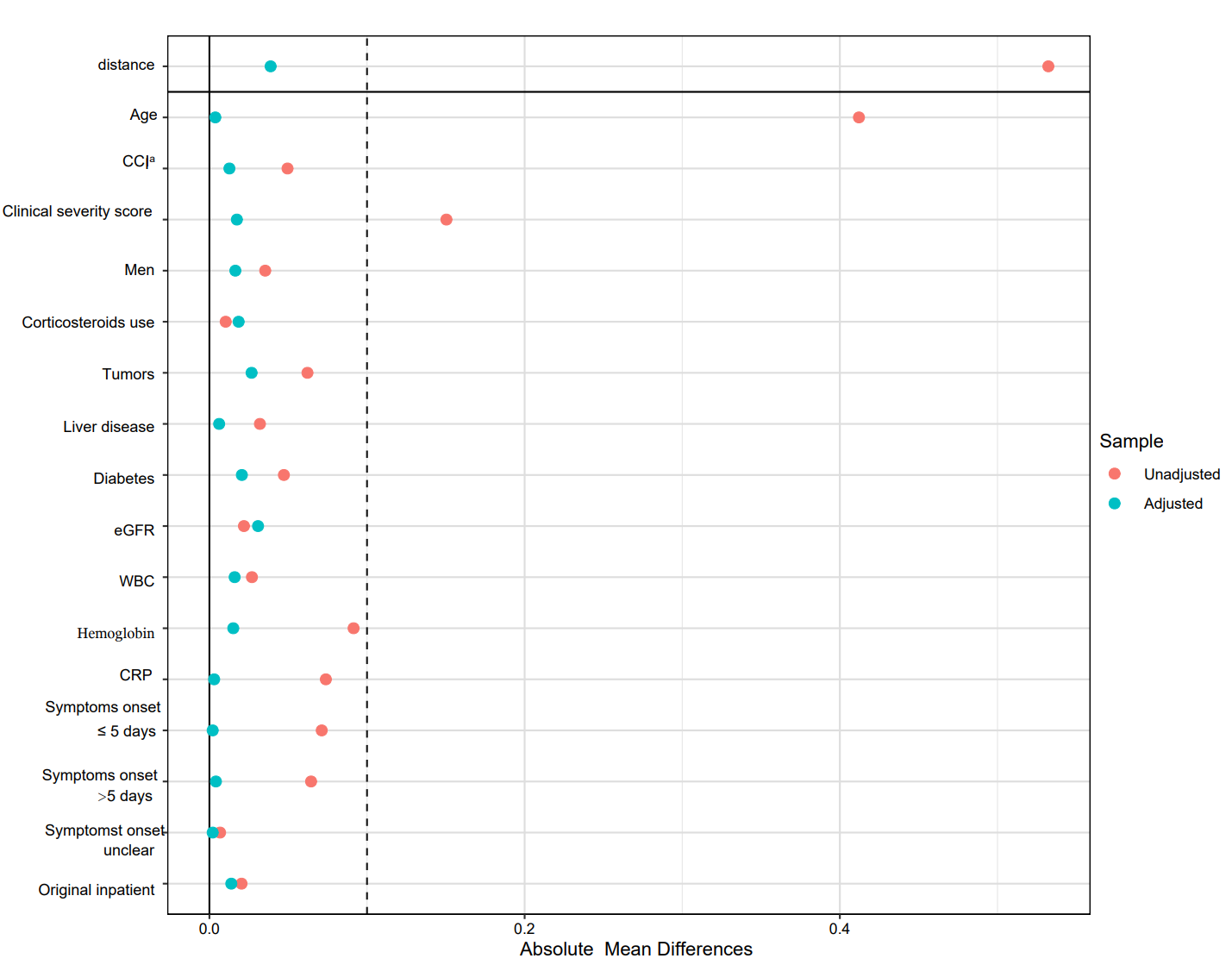
**

**Supplemental Figure 2. Love plot assessing differences in baseline characteristics before and after 1:1 propensity-score matching.**

**
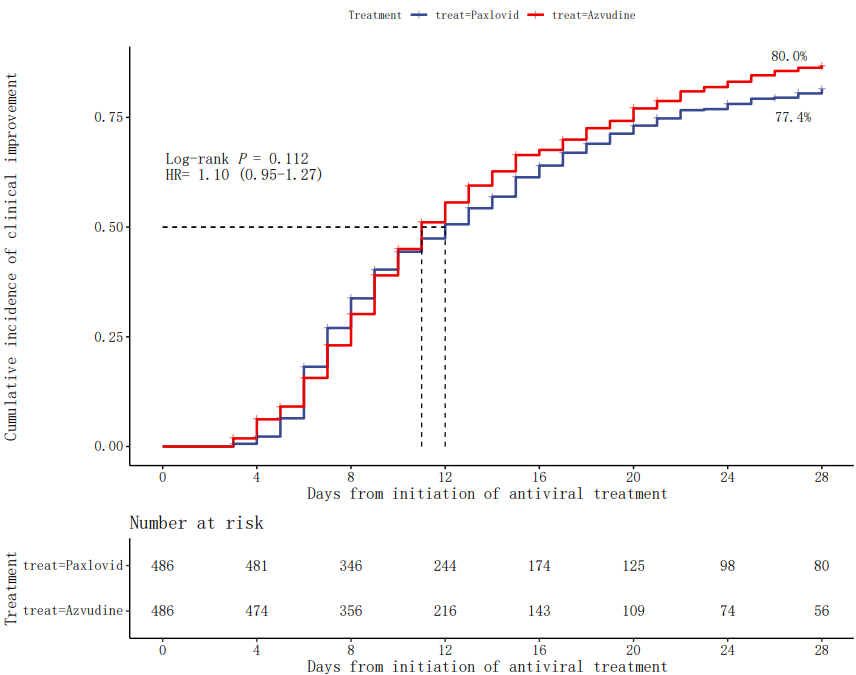
**

**Supplemental Figure 3. Cumulative incidence of clincal improvement events for nirmatrelvir–ritonavir（Paxlovid） group vs azvudine group.**

**
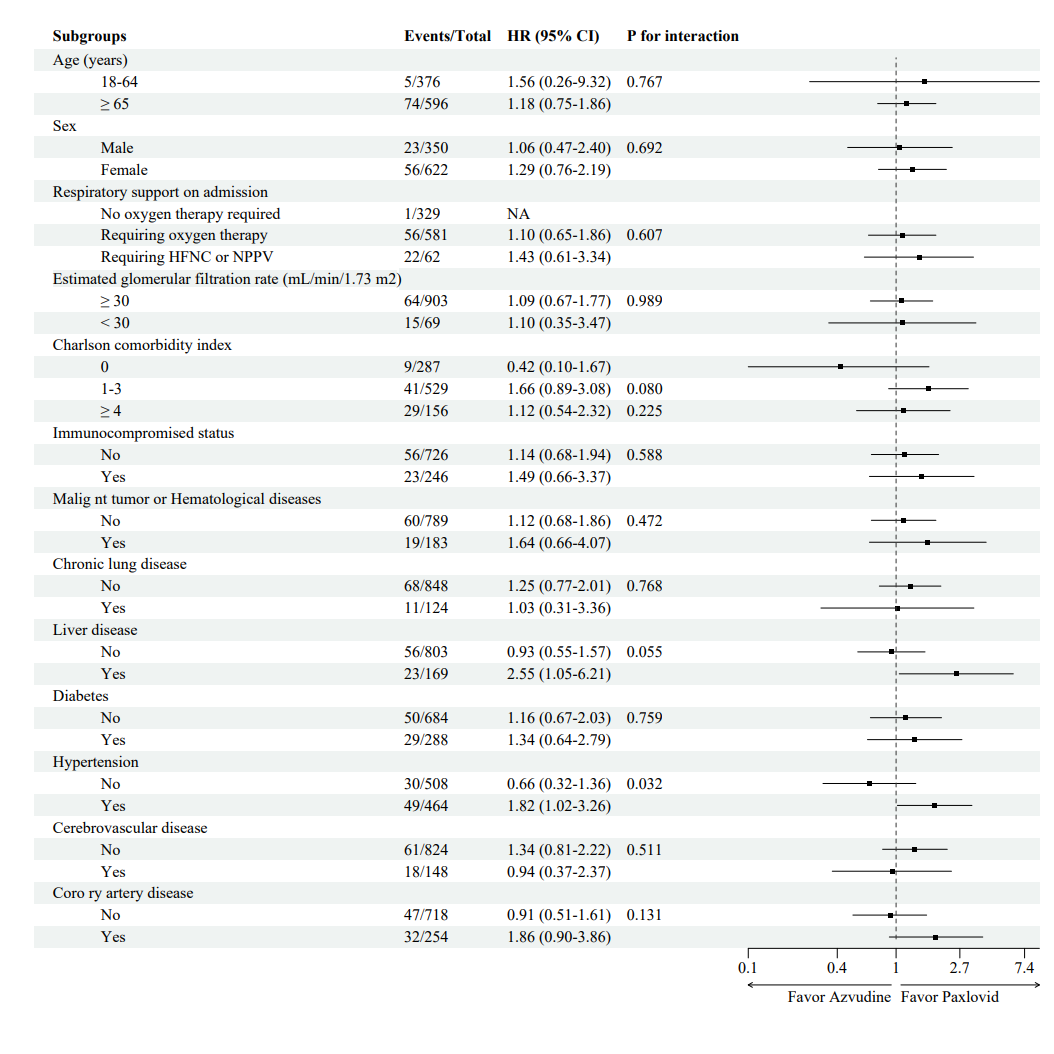
**

**Supplemental Figure 4. Subgroup analysis comparing all-cause mortality risks between the nirmatrelvir–ritonavir (Paxlovid) group and matched controls, as defined according to baseline characteristics.**

**
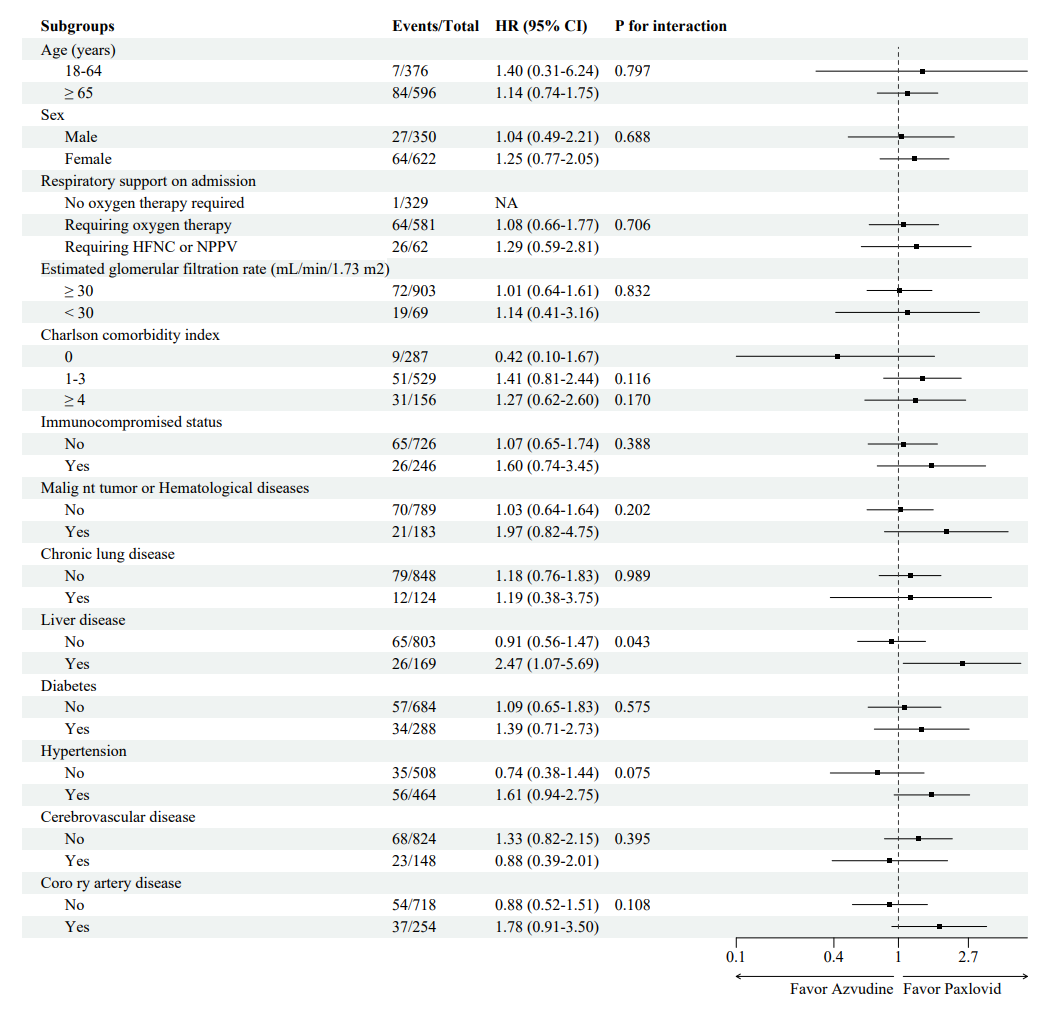
**

**Supplemental Figure 5. Subgroup analysis comparing incidences of composite disease progression between the nirmatrelvir–ritonavir (Paxlovid) group and azvudine group, as defined according to baseline characteristics**
